# Beyond clozapine – what options do we have in clozapine resistant schizophrenia?

**DOI:** 10.1192/j.eurpsy.2025.2236

**Published:** 2025-08-26

**Authors:** A. C. Matias, T. Vieira, A. Ferreira Silva, R. Machado Lopes, C. Almeida Rodrigues, F. Marinho Santos, P. Fonseca Coelho

**Affiliations:** 1 Psychiatry and Mental Health Department, Unidade Local de Saúde do Médio Tejo, Tomar, Portugal

## Abstract

**Introduction:**

Schizophrenia is one of the most disabling mental disorders, affecting around 1% of the population. Although most patients respond to antipsychotic treatment, one third have a limited response to antipsychotic medication, being considered treatment resistant schizophrenia (TRS). Clozapine is the only effective medication for TRS, but 30-40% of TRS patients do not respond to it. Patients with schizophrenia who do not respond to clozapine experience more severe disability, persistent symptoms, a lower quality of life and incur higher economic costs compared to those who respond to treatment.

**Objectives:**

The aim of the present study is to review evidence for therapeutic strategies for patients with treatment-resistant schizophrenia not responding to clozapine.

**Methods:**

Review of the literature regarding treatment-resistant schizophrenia not responding to clozapine. The research was carried out through the PubMed® database, using the terms “treatment resistant schizophrenia”, “schizophrenia resistant to clozapine” and “clozapine augmentation”.

**Results:**

For patients with treatment-resistant schizophrenia who do not respond to clozapine, several therapeutic strategies have been explored. These include pharmacological approaches, non-pharmacological interventions and brain stimulation procedures [Electroconvulsive Therapy (ECT) and Transcranial Magnetic Stimulation (TMS)]. However, the evidence is weak and the reported benefits were modest.

**Conclusions:**

The current evidence is weak for efficacy of pharmacological augmentation strategies to clozapine. There are contradictory data regarding ECT and clozapine augmentation. More studies are necessary to clarify the potential of these strategies in order to manage these complex patients.

**Disclosure of Interest:**

None Declared

